# Cardiovascular Effect of Incretin-Based Therapy in Patients with Type 2 Diabetes Mellitus: Systematic Review and Meta-Analysis

**DOI:** 10.1371/journal.pone.0153502

**Published:** 2016-04-14

**Authors:** Je-Yon Kim, Seungwon Yang, Jangik I. Lee, Min Jung Chang

**Affiliations:** 1 Department of Pharmaceutical Medicine and Regulatory Science, Colleges of Medicine and Pharmacy, Yonsei University, Incheon, Republic of Korea; 2 Department of Pharmacy and Yonsei Institute of Pharmaceutical Sciences, College of Pharmacy, Yonsei University, Incheon, South Korea; 3 College of Pharmacy and Research Institute of Pharmaceutical Sciences, Seoul National University, Seoul, South Korea; Baylor College of Medicine, UNITED STATES

## Abstract

**Background:**

To assess the cardiovascular (CV) risk associated with the use of incretin-based therapy in adult patients with type 2 diabetes mellitus (T2DM) primary prevention group with low CV risks.

**Methods:**

The clinical studies on incretin-based therapy published in medical journals until August 2014 were comprehensively searched using MEDLINE, EMBASE and CENTRAL with no language restriction. The studies were systemically reviewed and evaluated for CV risks using a meta-analysis approach and where they meet the following criteria: clinical trial, incidence of predefined CV disease, T2DM with no comorbidities, age > 18 years old, duration of at least 12 weeks, incretin-based therapy compared with other antihyperglycaemic agents or placebo. Statistical analyses were performed using a Mantel-Haenszel (M-H) test. The odds ratios (OR) and their 95% confidence interval (CI) were estimated and displayed for comparison.

**Results:**

A total of 75 studies comprising 45,648 patients with T2DM were selected. The pooled estimate demonstrated no significance in decreased CV risk with incretin-based therapy versus control (M-H OR, 0.90; 95% CI, 0.81–1.00).

**Conclusions:**

This meta-analysis suggests that incretin-based therapy show no significant protective effect on CV events in T2DM primary prevention group with low CV risks. Prospective randomized controlled trials are required to confirm the results of this analysis.

## Introduction

Type 2 diabetes mellitus (T2DM) is a chronic and progressive disease associated with both microvascular and macrovascular complications [1]. The risk of cardiovascular (CV) disease is known to be higher in people with diabetes compared to those without diabetes [2] and CV disease accounts for excess mortality in T2DM [3].

In the assessment of CV risks, glycated hemoglobin control was conventionally thought as related to CV risk owing to the United Kingdom Prospective Diabetes Study (UKPDS) 10-year follow-up study. The study demonstrated a significant reduction in myocardial infarction (MI) and all-cause mortality in overweight newly diagnosed patients with T2DM in intensive glycemic control with metformin [4]. Stemming from these results, improved glycemic control has been traditionally thought to reduce the risk of the microvascular complications of diabetes.

However, more recently, the Action in Diabetes and Vascular Disease: Preterax and Diamicron Modified Release Controlled Evaluation (ADVANCE) and the Veterans Affairs Diabetes Trial (VADT) did not find significant beneficial effects of intensive glucose control in nonfatal MI, nonfatal stroke, and overall CV mortality [5, 6]. Taken together, the results from clinical trials introduced controversy about the effect of glycemic control on CV disease risk, and uncertainty remains regarding whether any particular glucose lowering strategy actually lowers CV risk.

A recent perspective article published in *New England Journal of Medicine* by the US Food and Drug Administration (FDA) advisory committee members stated that the optimal approach to the reduction of cardiovascular risk in diabetes patients should focus on the management of standard cardiovascular risk factors rather than intensive glycemic control.[7]

From a drug safety perspective, there has been increasing concern and need of assurance regarding antihyperglycemic agents’ cardiovascular safety. After the concerns raised in 2008 about the cardiac safety of rosiglitazone, the FDA issued an updated Guidance for Industry that required pre and post approval studies to rule out excess cardiovascular risk of any new antidiabetic drug. [8].

In four previous CV trials on incretins [9–12], there was no evidence of an increase or decrease in the number of major adverse cardiovascular events but there were safety concerns regarding a possible elevated risk in hospitalization for heart failure.

Hence, there is a need for a rigorous evaluation of the cardiovascular safety of GLP-1 receptor agonists and DPP-4 inhibitors. In the absence of head-to-head trials, this analysis may provide valuable insight into the comparative outcomes of incretin overall class versus placebo or active control.

As a part of this study, we conducted a systematic review of randomized and controlled studies to provide a comprehensive assessment regarding the risk of cardiovascular diseases associated with DPP-4 inhibitors and GLP-1 receptor agonists compared to placebo or other antihyperglycaemic agents.

## Materials and Methods

### Data sources and searches

We conducted a search in MEDLINE (via PubMed), EMBASE, and the Cochrane Central Register of Controlled Trials (CENTRAL) up to August 2014. We developed a search strategy using MeSH and free text terms. Study type was restricted to randomized controlled trials, controlled trials, clinical trial, controlled clinical trial, controlled studies and clinical studies in humans.

### Study selection

We included studies that (1) enrolled adult patients (of at least 18 years of age) with T2DM with no other complications, (2) compared DPP-4 inhibitors or GLP-1 receptor agonists against placebo (placebo-controlled) or other antihyperglycemic agents (active-controlled), (3) duration of at least 12 weeks, and (4) had explicit reported events of predefined CV outcomes. Trials with shorter duration were excluded because of inadequate time to assess changes in glycemic efficacy, since hemoglobin A1_c_ reflects glycemia during previous 3 months [13].

We followed systematic approach to only include studies with patients who have no other complications at baseline in order to target the study group as primary prevention population and compare the CV effect of incretin in this patient group who are low CV risk patients without significant cardiovascular disease comorbidities or significant laboratory changes. To be classified as T2DM with no other complications, we ensured that the patients included had no underlying diseases at baseline. We also collected information on CV and renal biomarkers such as systolic blood pressure (SBP), diastolic blood pressure (DBP), HDL (high density lipoprotein) cholesterol, LDL (low density lipoprotein) cholesterol, total cholesterol (TC), triglycerides (TG), creatinine clearance (CrCl), serum creatinine (SCr), and glomerular filtration rate (GFR); we reviewed baseline level of each biomarker to exclude any above normal results. We excluded patients with baseline hypertension (SBP ≥ 140 mmHg or DBP ≥ 90 mmHg [14]) or history of hypertension or an antihypertensive treatment, dyslipidemia (HDL < 40 mg/dL, LDL > 130 mg/dL, TC > 200 mg/dL, TG > 150 mg/dL [15]), impaired renal function (CrCl < 30 mL/min, SCr > 1.2 mg/dL, GFR < 30 mL/min[16]) or history of renal disease of disease treatment, and history of other vascular diseases or disease treatment.

The predefined CV outcomes were classified as described below. This classification was reviewed by a cardiologist.

1. Death: cardiac death, sudden death, all causes of death.
2. Heart failure: heart failure, cardiac failure, cardiac myopathy.
3. Hypertension: hypertension, blood pressure change, hypertensive crisis.
4. Vascular disorders: dyslipidemia, hyperlipidemia, stroke, thrombosis, deep vein thrombosis, arteriosclerosis, raised triglycerides, raised LDL, decreased HDL, lipidemia, hypercholesterolemia, aortic valve sclerosis.
5. Coronary artery disease: angina, myocardial infarction, ischemia, revascularization, acute coronary syndrome, coronary artery blockage, ST elevation myocardial infarction, non-ST elevation myocardial infarction coronary artery stenosis, coronary artery disease.
6. Arrhythmia: arrhythmia, tachycardia, bradycardia, atrial fibrillation, atrial flutter, ventricular fibrillation, ventricular flutter, cardio-respiratory arrest, palpitation, ventricular extra systoles, supraventricular extra systoles, left bundle branch block.
7. Other/ Non-specified: chest pain, hypotension, cardiomegaly, cerebral infarction, syncope.

### Data extraction and quality assessment

Publications retrieved from three search engines were imported into the reference management software (Endnote^®^ X6, X7; Thomson Reuters, New York, NY). After removing duplicate results, two reviewers (KJY, CMJ) independently screened all titles, abstracts, and full texts according to the study process. Any discrepancies were resolved by discussion and adjudication by the third reviewer (YSW).

For quality assessment, we used Cochrane Collaboration’s tool [17] to assess the risk of bias of the included trials. We considered random sequence generation, allocation concealment, blinding of participants and personnel, blinding of outcome assessment, and incomplete outcome data and evaluated whether the adjudication of CV events was carried out. Risk of bias was evaluated at three levels: ‘low (low risk of bias)’, ‘high (high risk of bias)’ and ‘unclear’. The result was not used as a criterion for the selection of trials, but only for descriptive purposes.

To assess possible publication or disclosure bias we used funnel plots, the Begg adjusted rank correlation test [18]. Asymmetry in a funnel plot (also known as small study effects [19]) is potentially indicative of publication biases.

All analyses were performed using Review Manager 5.3 software (RevMan version 5.3; Copenhagen: The Nordic Cochrane Centre, The Cochrane Collaboration).

### Data synthesis

We further collected information from the selected trials on study characteristics (study design, study duration, total study population, total safety population), baseline patient characteristics (mean age, percentage of male, mean duration of diabetes, mean body mass index [BMI], mean fasting plasma glucose [FPG]), interventions (incretin treatment, control treatment, dose, drug used across groups, mode of therapy), duration of treatment and number of CV events reported.

For extension trials, we used data from the longest follow-up. If treatment assignments were exchanged or both arms were assigned to incretin therapy in the extension period, we collected data before that point, if provided.

Trials were excluded if intervention and comparator groups were both based on incretin therapy with no placebo or other antihyperglycemic group (e.g. sitagliptin vs. exenatide trial). If placebo and other antihyperglycemic agent group were both included or multiple incretin arms were included in the study, we reconstructed the study arms into incretin vs. comparator arm.

Add-on therapies included other oral antihyperglycemic agents (i.e., sulphonylureas, thiazolidinediones and biguanides) and injectable therapies (i.e., insulin) co-administered with the incretin-based therapies or the comparator arm (combination therapy). If there was at least one arm treated with a combination therapy, trial was classified as add-on therapy trial.

Active controlled studies were those compared with other antihyperglycaemic agents and placebo controlled studies were trials where comparator arm was placebo.

### Data analysis

We assessed heterogeneity between studies using the χ^2^ test and I^2^ statistic with a significance threshold for α of 0.10 [20]. We report the results of fixed-effect model because generally the heterogeneity data was not present in the studies included. We conducted a sensitivity analysis using an alternative heterogeneity consideration model on those subgroup analyses with high heterogeneity. We pooled trials using the Mantel-Haenszel method, since the number of CV events is dichotomous variables, Mantel-Haenszel odds ratio (MH-OR) with 95% confidence interval was calculated for all CV events defined. We performed a primary analysis to find out the overall incretin effect on predefined general CV risk. Furthermore, diverse subgroup analyses were performed: type of incretin (DPP-4 inhibitors vs. control, GLP-1 receptor agonist vs. control); type of control with mode of therapy (placebo controlled in mono therapy, placebo controlled in add-on therapy, active controlled in mono therapy, active controlled in add-on therapy); classified CV outcomes; and individual incretin agents.

## Results

**Fig 1:**
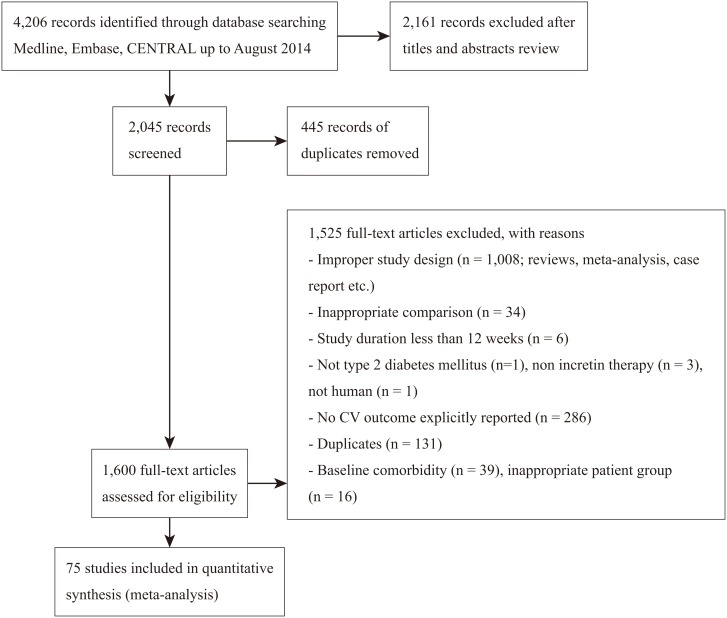
Flow chart of article selection (PRISMA flow diagram).

Our search yielded 4,206 potentially relevant reports. After screening titles and abstracts, we retrieved 1,600 reports for full text screening. A total of 75 studies were eligible for final inclusion comprising 45,648 patients (Fig 1). The median duration of the 75 trials—all industry funded—was 35 weeks (ranging from: 12 to 112 weeks). The trials enrolled a mean of 608 (ranging from 36 to 3,118 patients), and the population for safety analysis accounted for a mean of 610 patients (ranging from 36 to 3,099). The mean age was 56.1 years old and 54.1% were males. The mean BMI was 30.3 kg/m^2^ (ranging from 24.1 to 33.9 kg/m^2^), the mean baseline HbA1_c_ was 8.3% (ranging from 6.6 to 11.4%), the mean FPG was 9.5 mmol/L (ranging from 6.9 to 12.2 mmol/L), and the mean duration of diabetes was 6.5 years (ranging from 1.3 to 12.6 years). The average value was calculated excluding those not reported (Table 1).The intervention characteristics and number of CV events reported in each trial are summarized in Table 2. Fifty-eight trials tested DPP-4 inhibitors, 16 tested GLP-1 receptor agonists, and one tested both agents (Table 2).

**Table 1:** Characteristics of included trials of incretin treatment in patients with type 2 diabetes mellitus.

Author (year)	Total population	Safety population	Study duration (weeks)	Mean Age (years)	Male (%)	Mean diabetes duration (years)	Mean BMI (kg/m^2^)	Mean HbA1C (%)	MeanFPG (mmol/L)
Aschner 2006[37]	741	741	24	54.2	51.7	4.4	30.5	8.0	9.7
Aschner 2010[38]	1,050	1,050	24	56.0	46.0	2.4	30.8	7.2	7.9
Barnett 2012[39]	455	455	24	57.2	41.7	11.9	32.3	8.7	9.6
Bergenstal 2009[40]	372	372	24	52.6	48.1	9.0	33.8	10.2	11.3
Blonde 2009[41]	2,664	2,627	12	55.6	51.8	5.1	32.4	8.0	9.3
Bolli 2009[42]	576	576	52	56.6	62.9	6.4	32.2	8.4	10.9
Bosi 2007[43]	416	541	24	54.2	57.4	6.3	32.7	8.4	9.9
Bosi 2009[22]	1,179	1,171	24	52.8	58	2.0	31.2	8.7	10.4
Chacra 2011[44]	768	768	76	55.1	45.1	6.9	29	8.4	9.6
Del 2011[45]	503	503	24	55.7	48.3	NR	29.1	8	8.9
Dobs 2013[46]	262	260	54	54.5	54.8	9.3	30.3	8.8	10.1
Filozof 2010[47]	1,007	1,007	52	59.5	52	6.6	31	8.5	10.7
Fonesca 2012(2)[48]	361	361	12	53.7	51.5	1.3	31.9	8.0	9.0
Fonseca 2012[49]	282	282	18	55.4	46.1	6.2	30.9	8.3	9.0
Fonseca 2013[50]	313	313	26	56.0	62.3	9.8	29.9	8.8	9.8
Forst 2010[51]	333	333	12	60.0	58	7	31.9	8.3	10.3
Gallwitz 2012[52]	1,029	1019	48	56.0	54.0	5.7	32.5	7.5	8.8
Gallwitz 2012(2)[23]	1,551	1551	104	59.8	60.5	715(47.1%)*	30.2	7.7	9.1
Garber 2007[53]	398	462	24	54.3	50.0	4.7	32.4	8.7	10.1
Garber 2008[54]	408	515	24	58.2	59.0	7.2	31.3	8.5	10.4
Garber 2011[55]	745	745	104	53.0	50.0	5.4	33.1	8.3	9.4
Goke 2013[56]	858	858	104	57.6	51.8	5.4	31.4	7.7	9.0
Goodman 2009[57]	370	370	24	54.8	57.5	NR	31.5	8.6	10.9
Grunberge 2012[58]	164	164	12	56.6	45.1	3.9	32.1	7.2	NR
Haak 2012[59]	791	791	24	55.3	53.8	562(74.3%)*	29.1	8.7	10.8
Henry 2011[21]	36	36	12	55.6	38.9	3.1	32.9	6.8	7.1
Hollander 2011[60]	565	565	76	54.0	49.6	5.2	30.0	8.3	9.0
Inagaki 2012[61]	427	427	26	56.8	67.9	9	26.2	8.5	9.0
Inagaki 2014[62]	322	322	12	59.8	60.3	6.4	25.3	8.1	9.1
Iwamoto 2010[63]	363	363	12	59.8	61.7	5.4	24.5	7.6	8.2
Kaku 2011[64]	339	339	52	60.1	62.8	6.7	26.1	7.9	NR
Kaku 2011(2)[65]	400	400	52	58.3	67.3	8.3	24.8	9.3	NR
Matthews 2010[66]	3,118	3,099	104	57.5	53.5	5.7	31.8	7.3	9.2
Matyjaszek-Matuszek 2013[67]	80	80	26	60	43.8	8.4	32.1	7.9	9.6
Mohan 2009[68]	530	530	18	50.9	58.0	2.0	25.0	8.7	10.5
Moses 2014[69]	257	257	24	57.0	59.9	NR	29.3	8.3	8.8
Nauck 2007[70]	1,172	1,172	52	56.7	59.2	6.4	31.2	7.7	9.2
Nauck 2009[71]	527	527	26	54.8	50.3	6	32	302(57.3%)**	9.5
Nauck 2013[24]	1,091	1,087	104	56.7	58.2	7.6	31	8.4	10
Nonaka 2008[72]	151	151	12	55.3	63	4	25.2	7.6	9.1
Olansky 2011[73]	1,246	1,246	44	49.7	56.5	3.4	33.3	9.9	10.3
Perez-Monteverde 2011[74]	492	452	40	51.1	61.0	3.2	29.8	9.1	10.3
Pfutzner 2011[75]	1,306	1,306	76	52.0	49.2	1.7	30.4	9.5	11.1
Phillis-Tsimikas 2013[76]	447	454	26	40.8	58.6	7.8	30.4	8.9	9.6
Pinget 2013[77]	484	484	24	55.8	52.3	8.1	33.9	8.1	9.1
Pratley 2006[78]	98	98	12	55.7	42.9	4.3	30.0	8.0	9.6
Pratley 2009[79]	500	500	26	56.6	52.2	7.7	30.1	8.1	NR
Pratley 2009(2)[80]	493	493	24	55.4	58.2	7.6	32.8	8.0	NR
Pratley 2013[81]	760	751	24	56.4	49	8.8	32.7	8.3	10.0
Prato 2011[82]	503	503	24	55.7	48.3	NR	29.1	8	7.1
Ratner 2010[83]	129	129	12	57	14.0	7.0	32.4	7.9	9.1
Raz 2008[84]	190	190	30	54.8	46.3	7.9	30.2	9.2	11.1
Reasner 2011[85]	1,246	1,246	18	49.7	56.5	3.4	33.3	9.9	12.2
Riddle 2013[86]	495	495	24	57	46	12.5	32.1	8.4	8.0
Rosenstock 2006[87]	353	353	24	56.3	55.5	6.1	31.5	8.0	9.3
Rosenstock 2009[88]	598	591	80	54.3	56.6	2.2	32.6	8.6	9.9
Rosenstock 2009(2)[89]	390	390	26	56.8	41.4	12.6	32.5	9.3	10.6
Rosenstock 2011[90]	859	859	24	57.3	50.5	9.3	30.2	8.3	9.5
Ross 2012[91]	491	491	12	58.6	57	227(46.2%)*	29.6	7.9	9.2
Russel-Jones 2012[92]	820	820	26	53.8	59.0	2.7	31.2	8.5	9.9
Scherbaum 2008[93]	131	131	112	63.1	59.5	2.3	30.3	6.6	6.9
Schernthaner 2013[94]	755	755	52	56.7	55.9	9.6	31.6	8.1	9.3
Seino 2012[95]	312	312	64	60.2	66.7	9.8	24.7	8.6	NR
Seino 2012(2)[96]	288	288	12	52.6	68.8	6.3	25.9	8	NR
Seino 2014[97]	215	212	16	57	69.8	7	25.1	8.6	NR
Strain 2013[98]	278	278	24	74.8	45.3	11.4	29.8	11.4	9.8
Tajima 2011[99]	138	138	12	60.8	58	9.1	24.6	8.4	8.6
Tajima 2013[100]	133	133	12	60.5	65.4	7.2	24.1	7.9	8.4
Taskinen 2011[101]	700	700	24	56.5	54	310(44.3%)*	29.9	8.1	9.4
Terra 2011[102]	301	301	12	56.2	66.4	7.1	32.0	8.3	9.5
Vilsboll 2010[103]	641	641	24	57.8	51.0	12.5	31.0	8.7	9.8
White 2014[104]	160	160	12	55.4	53.1	6.0	33.1	7.9	9.1
Williams-Herman 2009[105]	1,091	1,091	54	53.5	50.6	4.5	32.1	8.8	11.1
Wysham 2014[106]	976	976	26	55.6	58.4	9.0	33.3	8.1	9.0
Yang 2011[107]	570	570	24	54.1	48.3	5.1	26.2	7.9	8.8
**Average value across included trials**	**608**	**610**	**35.1**	**56.1**	**54.1**	**6.5**	**30.3**	**8.3**	**9.5**

BMI = body mass index; FPG = fasting plasma glucose; NR = not reported

* No (%) of patients with no more than 5 years’ diabetes duration

** No (%) of patients with HbA1_c_ < 8%

**Table 2:** Intervention characteristics of included trials of incretin treatment in patients with type 2 diabetes mellitus.

Author (year)	Incretin		Control		Drugs used across groups	Mode of therapy
	type	CV event	type	CV event		
Aschner 2006	sitagliptin	14/488	placebo	6/253	none	mono
Aschner 2010	sitagliptin	12/528	metformin	4/522	none	mono
Barnett 2012	saxagliptin	1/304	placebo	0/12	insulin ±metformin	add on
Bergenstal 2009	exenatide	0/124	biphasic insulin aspart	1/248	none	mono
Blonde 2009	vildagliptin	5/1756	thiazolidinediones	1/871	metformin	add on
Bolli 2009	vildagliptin	2/296	pioglitazone	6/280	metformin	add on
Bosi 2007	vildagliptin	6/360	placebo	3/181	metformin	add on
Bosi 2009*	vildagliptin	17/879	metformin	12/292	metformin	add on
Chacra 2011	saxagliptin	92/501	placebo	45/267	glyburide	add on
Del 2011	linagliptin	21/336	placebo	6/167	none	mono
Dobs 2013	sitagliptin	2/170	placebo	0/90	metformin +rosiglitazone	add on
Filozof 2010	vildagliptin	36/510	gliclazide	43/493	metformin	add on
Fonesca 2012(2)	lixisenatide	1/239	placebo	0/122	none	mono
Fonseca 2012^†^	saxagliptin	1/238	metformin	2/144	metformin	add on
Fonseca 2013	sitagliptin	0/157	placebo	1/156	metformin +pioglitazone	add on
Forst 2010	linagliptin	3/197	placebo, glimepiride	0/136	metformin	add on
Gallwitz 2012	exenatide	0/511	glimepiride	4/508	metformin	add on
Gallwitz 2012(2)	linagliptin	62/776	glimepiride	86/775	metformin	add on
Garber 2007	vildagliptin	1/304	placebo	1/158	pioglitazone	add on
Garber 2008	vildagliptin	6/339	placebo	1/176	glimepiride	add on
Garber 2011	liraglutide	59/497	glimepiride	34/248	none	mono
Goke 2013	saxagliptin	20/428	glipizide	32/430	metformin	add on
Goodman 2009	vildagliptin	5/248	placebo	3/122	metformin	add on
Grunberge 2012	dulaglutide	1/132	placebo	0/32	none	mono
Haak 2012*	linagliptin	0/428	placebo/metformin	1/429	metformin	add on
Henry 2011	saxagliptin	0/20	placebo	0/16	none	mono
Hollander 2011	saxagliptin	36/381	placebo	14/184	thiazolidinediones	add on
Inagaki 2012	exenatide	1/215	insulin glargine	0/212	biguanide ±thiazolidinedione	add on
Inagaki 2014	SYR-472 (DPP-4 inhibitor)	10/266	placebo	3/55	none	mono
Iwamoto 2010	sitagliptin	3/290	placebo	0/73	none	mono
Kaku 2011	alogliptin	2/224	placebo	1/115	pioglitazone	add on
Kaku 2011(2)	liraglutide	34/268	glibendamide	24/132	none	mono
Matthews 2010	vildagliptin	104/ 1553	glimepiride	125/ 1546	metformin	add on
Matyjaszek-Matuszek 2013	exenatide	2/40	insulin glargine	0/40	metformin +sulfonylurea	add on
Mohan 2009	sitagliptin	1/352	placebo	0/178	none	mono
Moses 2014	saxagliptin	12/129	placebo	9/128	metformin +sulfonylurea	add on
Nauck 2007	sitagliptin	0/588	glipizide	2/584	metformin	add on
Nauck 2009	alogliptin	14/423	placebo	7/104	metformin	add on
Nauck 2013	liraglutide	68/724	glimepiride/ placebo	14/363	metformin	add on
Nonaka 2008	sitagliptin	0/75	placebo	2/76	none	mono
Olansky 2011^¥^	sitagliptin	0/625	placebo	1/621	metformin other OHA	add on
Perez-Monteverde 2011**	sitagliptin	1/222	pioglitazone	0/230	metformin	add on
Pfutzner 2011*	saxagliptin	71/978	metformin	27/328	metformin	add on
Phillis-Tsimikas 2013	sitagliptin	0/228	insulin degludec	1/226	metformin	add on
Pinget 2013	lixisenatide	0/323	placebo	1/161	pioglitazone± metformin	add on
Pratley 2006	vildagliptin	6/70	placebo	6/28	none	mono
Pratley 2009	alogliptin	18/401	placebo	2/99	glyburide	add on
Pratley 2009(2)	alogliptin	19/397	placebo	1/97	pioglitazone	add on
Pratley 2013	taspoglutide	4/494	pioglitazone	2/257	sulphonylurea ±metformin	add on
Prato 2011	linagliptin	21/336	placebo	6/167	none	mono
Ratner 2010	taspoglutide	0/97	placebo	1/32	metformin	add on
Raz 2008	sitagliptin	5/96	placebo	5/94	metformin	add on
Reasner 2011^¥^	Sitagliptin	6/625	placebo	10/621	metformin	add on
Riddle 2013	lixisenatide	1/328	placebo	0/167	basal insulin ± metformin	add on
Rosenstock 2006	sitagliptin	1/175	placebo	0/178	pioglitazone	add on
Rosenstock 2009	vildagliptin	46/393	rosiglitazone	27/198	none	mono
Rosenstock 2009(2)	alogliptin	1/260	placebo	0/129	insulin	add on
Rosenstock 2011	lixisenatide	1/574	placebo	0/285	sulfonulurea ± metformin	add on
Ross 2012	linagliptin	6/447	placebo	0/44	metformin	add on
Russel-Jones 2012^ǂ^	exenatide/ sitagliptin	6/411	pioglitazone/ metformin	15/409	none	mono
Scherbaum 2008	vildagliptin	6/68	placebo	2/63	none	mono
Schernthaner 2013	sitagliptin	0/378	canagliflozin	2/377	metformin +sulfonylurea	add on
Seino 2012	alogliptin	2/209	placebo	0/103	glimepiride	add on
Seino 2012(2)	alogliptin	3/188	placebo	2/100	metformin	add on
Seino 2014	albiglutide	4/159	placebo	1/53	none	mono
Strain 2013*	vildagliptin	6/139	placebo	3/139	sulphonylurea	add on
Tajima 2011	sitagliptin	0/67	placebo	2/71	glimepiride	add on
Tajima 2013	sitagliptin	1/70	placebo	0/63	voglibose	add on
Taskinen 2011	linagliptin	17/523	placebo	6/177	metformin	add on
Terra 2011	PF-734200 (DPP-4 inhibitor)	2/225	placebo	1/76	metformin	add on
Vilsboll 2010	sitagliptin	0/322	placebo	2/319	insulin ± metformin	add on
White 2014	saxagliptin	3/74	placebo	2/86	metformin	add on
Williams-Herman 2009*	sitagliptin	1/551	Placebo, metformin	1/540	metformin	add on
Wysham 2014	dulaglutide/exenatide	1/835	placebo	0/141	metformin + pioglitazone	add on
Yang 2011	saxagliptin	11/283	placebo	19/287	metformin	add on

*: Not all but some treatment groups were co-administered with metformin or sulfonylurea.

**: metformin was co-administered from the start of phase B

^†^: metformin was uptitrated in control group where we defined these treatment arms as add-on therapy, and mentioned metformin as drug used across groups

^ǂ^: Among 4 study groups in this study, we reconstructed the study population and lumped exenatide and sitagliptin group as ‘incretin’ and pioglitazone and metformin group as ‘non-incretin comparator’.

^¥^: incretin groups consisted fixed dose combination formulation with metformin

FDC: fixed-dose combination, OHA: oral hyperglycaemic agent

+: both A and B, -: either A or B, ±: A with or without B, /: A,B consists each separate arm

The heterogeneity between included trials was low according to both of the statistics tests we used (I^2^ = 2%, χ^2^ test = 74.12 [p = 0.44]). Seven studies had a prospective independent clinical event committee (CEC), which reviewed and adjudicated events suspected to be CV outcomes.

The shape of Begg’s funnel plot (S2 Fig) showed only minor asymmetry (with or without inclusion of the studies lacking individual participant data), and Egger’s test for asymmetry was not significant (P = 0.14). Thus a publication bias mechanism was not considered a major cause for concern in our study.

Of the 75 randomized controlled trials reporting predefined CV outcomes, only one study stated that no events of CV disease occurred during the course of study [21]. In total, 27,764 patients were recruited in intervention groups reporting 924 CV events (3.3%), and 17,884 patients were recruited in control group reporting 641 CV events (3.6%). Two out of 75 included trials, both with DPP-4 inhibitors, independently showed statistical significance in lowering CV risk [22, 23], and one GLP-1 receptor agonist study, in which liraglutide was added to metformin, showed statistical significance in increased CV risk [24].

**Fig 2:**
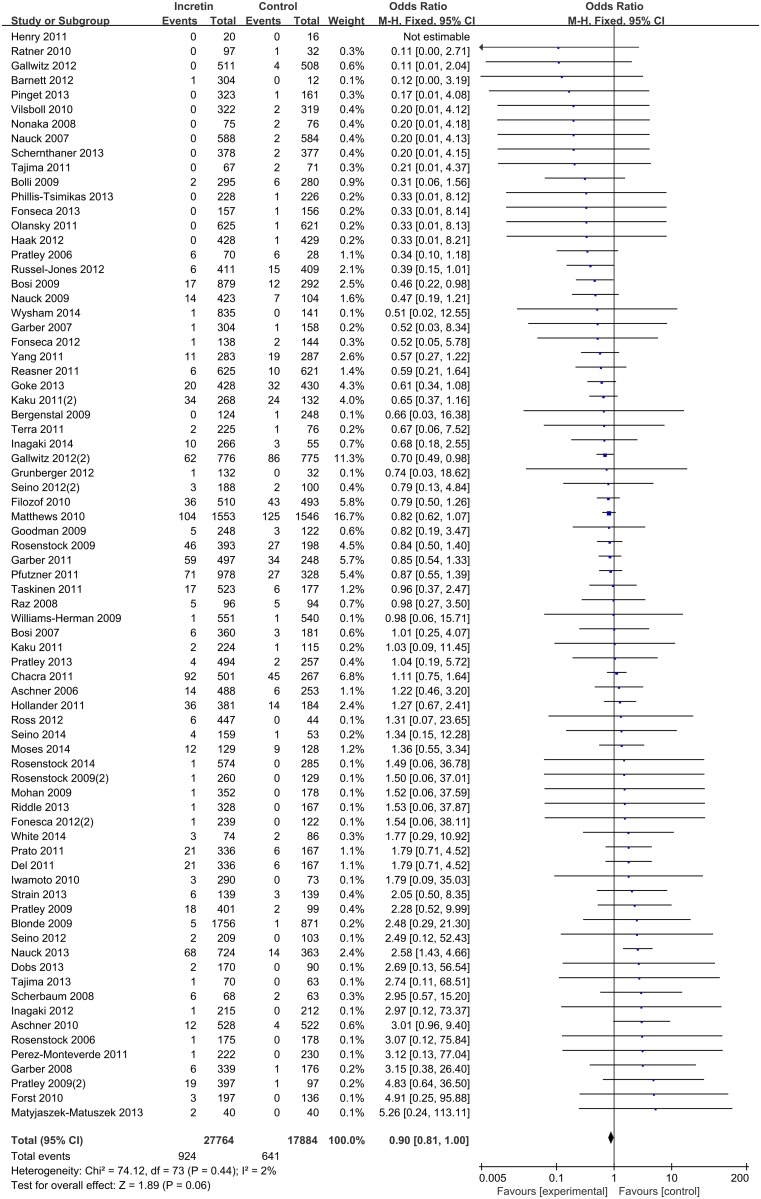
Risk of cardiovascular events between patients with type 2 diabetes mellitus with no other complications treated with incretin or control.

**Fig 3:**
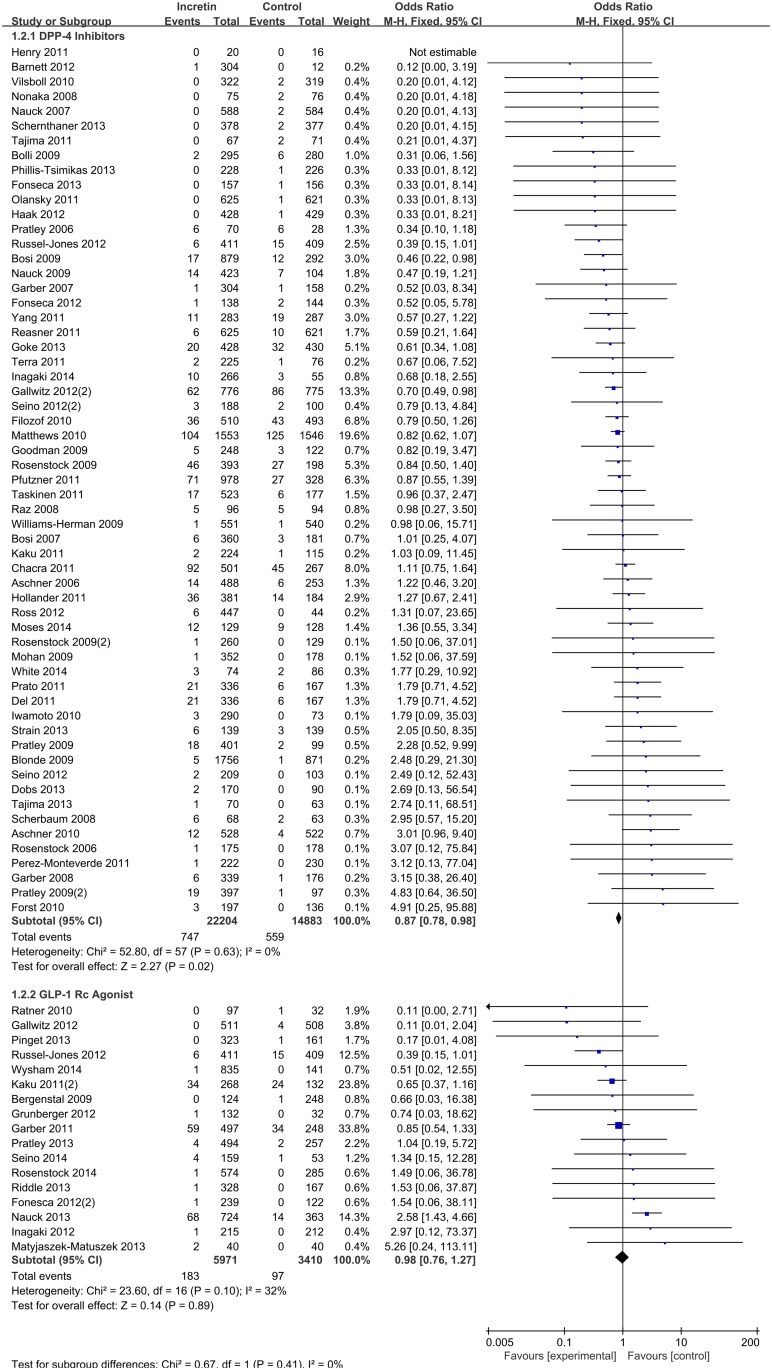
Subgroup analysis by types of incretin therapy.

In our primary analysis, pooled estimates of 75 studies showed no significance in beneficial effect of all incretin versus control (M-H OR 0.90, 95% CI 0.81–1.00) on CV risk (Fig 2). Evaluated as a subgroup, DPP-4 inhibitors alone were mildly protective compared to control (M-H OR 0.87, 95% CI 0.78–0.98), whereas the subgroup including only GLP-1 agonist showed no evidence of protection (M-H OR 0.98, 95% CI 0.76–1.27) (Fig 3) (χ^2^ test for subgroup differences, p-value = 0.41).

Also when we explored the sources of heterogeneity by type of control and mode of therapy, incretin therapy showed beneficial effect in comparison to an active comparator in add-on therapy (M-H OR 0.83, 95% CI 0.71–0.96). Whereas no such effect was observed in placebo-controlled trials and active-controlled mono therapy trials (S3 Fig) (χ^2^ test for subgroup differences, p-value = 0.10).

In subgroup analysis by type of 8 predefined CV outcome, no significant effect of incretin was observed in any. In case of death and heart failure, there were many cells with zero events and the maximum number of events per arm was 3, resulting in a small number of total reports and a wide confidence interval (S4 Fig) (χ^2^ test for subgroup differences, p-value = 0.35).

The subgroup analysis by type of individual incretins did not show difference among those agents except for vildagliptin, of which showed CV protective effect (M-H OR 0.82,95% CI 0.68–0.99) (S5 Fig) (χ^2^ test for subgroup differences, p-value = 0.72).

After performing the subgroup analyses to detect heterogeneity, we found that only two variables in all separate analyses displayed high heterogeneity (p-value lower than 0.1): the ‘other/non-specified’ subgroup by classification of predefined CV outcome, and the ‘liraglutide’ subgroup by individual incretin agent analysis. We performed a sensitivity analysis on these two subgroups using random-effect model and the results were consistent.

## Discussion

### Main findings

This meta-analysis included a comprehensive search for all trials with incretin-based therapies (GLP-1 receptor agonists and DPP-4 inhibitors) for type 2 diabetes treatment. The wide-ranging search allowed separate analyses by type of incretin, type of control and mode of therapy, classification of CV outcome and individual incretin agent.

The overall heterogeneity was low, including all subgroup analyses performed in the present study, which allowed us to apply a fixed-effect model rather than a random-effect model for data pooling.

Overall, incretin-based therapies showed a trend towards lower risk of cardiovascular disease compared to placebo or other antihyperglycemic agents, although the difference was not statistically significant.

The subgroup analysis by type of incretin showed statistical significance between groups; the effect of DPP-4 inhibitors on overall reduction of CV risk was greater than that of GLP-1 receptor agonists. These results suggest that, although drugs may share the pharmacological mechanism of increasing incretin activity, they may have different effects on CV risks. Contrary to our results, a retrospective analysis of the insurance claims database showed lower risk of CV events and hospitalizations in treatment with exenatide twice daily therapies than other-glucose lowering therapies [25].

Interestingly, incretin-based therapies were associated with a reduction of CV risk when compared to active antihyperglycemic agents treated in add-on therapies, whereas this effect disappeared when including placebo-comparator trials. Most of the trials in this group (79%) were DPP-4 inhibitors in combination with metformin compared to thiazolidinediones and sulfonylureas. It suggests incretin-based therapies added to metformin or other antihyperglycemic agents (as it is frequently used as second line therapy in treatment of T2DM) have beneficial effects on decreasing CV risks.

No additional effect was found in the subtypes of CV outcomes. Considering that we excluded a number of trials with baseline CV risks in patient characteristics, we infer that incretin-based therapies are associated with lower risk of major CV events, such as cardiac death and heart failure, in primary prevention group patients with low CV risk. Yet, as we collected safety outcomes reported from each study, which incorporates signs and symptoms that are not always a specific diagnosis, definitions of specific major CV events may have varied across studies.

In addition, three individual incretin agents, alogliptin, albiglutide, and liraglutide had higher odds of overall CV risk than comparators, but this difference was not statistically significant. However, vildagliptin was suggested to have a lower risk of CV disease with borderline significance (OR 0.82, 95% CI 0.68 to 0.99), which is consistent with the results from pooled data of 25 phase 3 trials assessing cardio-cerebrovascular safety of vildagliptin [26].

### Comparison with other studies

The available evidence regarding incretin-based therapies association with CV risk is currently contradictory. The reports indicate either a detrimental or a beneficial effect. Here we review several studies according to their study design.

Preclinical data indicated a potential cardio protective effect of DPP-4 inhibitors by increasing the concentration not only of GLP-1, but of other vasoactive peptides as well [27]. Some evidence shows that GLP-1 might have beneficial effects on the myocardium and on endothelial function [28] and GLP-1 has been found to be cardio protective in experimental models of heart failure and myocardial infarction [29].

Epidemiologic study data have shown the opposite results of our study, suggesting that exenatide reduces CV disease events (myocardial infarction, ischemic stroke, or coronary revascularization procedure) [25] and sitagliptin increases the risk of CV disease related hospital admissions and deaths [30]. But in both studies, when history of CV disease was measured, 50 to 60% of included patients with hypertension and dyslipidemia apart from our included patient group. And also both these studies were retrospective database analyses performed using insurance claim data, which are known to have substantial limitations such as misclassification of exposure and outcome using ICD codes mapping.

Patient level results from all completed phase 2/ 3 studies of liraglutide, alogliptin and vildaglipitin respectively showed no relevant significant effect on CV events [26, 31, 32].

Since US FDA now requires all new antidiabetic agents to undergo a thorough long-term CV risk assessment[8], recently four large-scale trials designed for this purpose in incretin have been carried out and currently there are many trials still ongoing expected to be published in forthcoming years (i.e CAROLINA, EXSCEL, LEADER and et cet.).

SAVOR TMI-53 included 16,492 patients with a history of, or at risk of CV events and they were randomly assigned to receive saxagliptin or placebo for an average of 2.1 years. The overall hazard ratio was 1.00 (95% CI 0.89–1.12), but the risk of hospitalization for heart failure was significant (hazard ratio 1.27, 95% CI 1.07–1.51) [9].

In the alogliptin trial (Examination of Cardiovascular Outcomes with Alogliptin vs. Standard of Care in Patients with Type 2 Diabetes Mellitus and Acute Coronary Syndrome, EXAMINE), where 5,380 patients were randomly assigned to receive alogliptin or placebo for a median of 18 months after an episode of acute myocardial infarction or unstable angina. The hazard ratio was 0.96 (upper boundary of the one-sided repeated confidence interval, 1.16) [33].

In the TECOS (cardiovascular outcomes trial of sitagliptin in T2DM), 14,671 patients were assigned to a group where either sitagliptin or placebo were added to their existing therapy, the median follow-up was 3 years. The trial achieved its primary endpoint of noninferiority for the composite CV endpoint of CV-related death, non-fatal myocardial infarction, non-fatal stroke, or unstable angina requiring hospitalization.—[11]. The observation that sitagliptin therapy was not associated with a change in long-term rates of cardiovascular events is consistent with the findings from shorter-term outcome trials of other DPP-4 inhibitors, including the above mentioned saxagliptin and alogliptin.

Last, but most recently, first GLP-1 receptor agonist CV outcome trial result was published which met the pre-specified criterion of non-inferiority versus placebo for the composite primary endpoint of CV death, non-fatal MI, non-fatal stroke and hospitalization for unstable angina (HR 1.02, 0.89–1.17) [12] (Lixisenatide CV outcome trial was published in December 2015. As our search includes records up to August 2014, this was not included in current meta-analysis. Only included in the discussion upon reviewer’s request).

In randomized controlled CV outcome trials, the patient inclusion criteria were history of established CV disease or multiple CV risk factors. All asserted such criteria because it is known that the risk of CV disease is 2 to 4 times higher in people with diabetes[34]. However, it is also known that diabetes substantially increases the risk of major CV complications with and without an established CV disease. Our study focused on the primary prevention patient group with low CV risk. This may explain the differences in results.

Two other meta-analyses have assessed the risk of CV disease among patients using incretins, both examining DPP-4 inhibitors. In the first meta-analysis, overall risk of acute heart failure was higher in patients treated with DPP-4 inhibitors compared to placebo or active comparators (M-H OR: 1.19 (95% CI 1.03–1.37) [35]. However, SAVOR TIMI-53 (Saxagliptin Assessment of Vascular Outcomes Recorded in patients with diabetes mellitus- Thrombolysis in Myocardial Infarction trial) trial [9] accounted for almost two thirds of all included events in this meta-analysis for heart failure, which raises question because SAVOR was large CV outcome trial resulting in safety concerns regarding possible elevated risk in hospitalization for heart failure. Therefore, this might have affected the results of our study suggesting a higher OR in the DPP-4 group.

Another DPP-4 inhibitor meta-analysis included 70 trials, enrolling 41,959 patients with a mean follow-up of 44.1 weeks. The MH-OR was 0.71 (95% CI 0.59–0.86), 0.64 (95% CI 0.44–0.94), 0.77 (95% CI 0.48–1.24) and 0.60 (95% CI 0.41–0.88) for major adverse cardiovascular events (MACE), myocardial infarction, stroke and mortality, respectively. Treatment with DPP4-inhibitors was suggested to reduce the risk of CV events (particularly myocardial infarction) and all-cause mortality in patients with T2DM [36]. The result showed a similar trend of lowering CV risk in DPP-4 inhibitors as our study but had statistical significance. This study had no further restrictions in baseline CV risk, and primary endpoint was only MACE and it also used clinicaltrials.gov as data source. These may have affected difference in included studies and difference in statistical significance.

The heterogeneity of results across meta-analyses or pooled analyses could depend on differences across studies in trial inclusion criteria, definition of events and event adjudication. With respect to pooled analyses of patient-level data, which are available only for each compound separately, the present meta-analysis has the advantage of integrating results for the whole class, thus increasing sample size and statistical power.

### Limitations

The primary limitation of this study is that the analysis was executed on summaries of trial results because original source data at patient-level were not available. This prevented the use of potentially more informative descriptions of events. Some of the adverse events reported were aggregated in the study result, for example, reported as non-specified CV disorder, vascular disease, etc. This may had effect in underestimating subgroup analysis of classified CV outcomes. Also, when screening patients with other comorbidities at baseline, we used patient baseline characteristics described in main result only. This practice may not have sufficiently ruled out trials with CV risk patients in the baseline. Similarly, we only included trials explicitly reporting a number of CV adverse events. Thus studies suggesting safety results without specific numbers for each treatment arm, such as ‘cardiovascular event was similar in both groups’ were omitted. In addition, we only gathered information on reported adverse events and we did not focus on the change of cardiovascular markers.

A further limitation is that it is possible that a publication bias affected this analysis, although the funnel plot did not show = significant asymmetry. We did not look for unpublished studies through other sources, so publication bias remains a relevant issue in this review.

Long term safety is of particular concern, the need for long term data on CV outcomes is especially important given the concerns with thiazolidinediones, but trials included had relatively short durations. Mean duration of included studies were 35 weeks, ranging from 12 to 112 weeks, but 70% of the trials included lasted for less than 30 weeks.

Most of the studies included were not primarily aimed at CV end-points. In addition, most trials did not centrally adjudicate CV outcomes. For this reason, a method for assessing CV events was not clearly specified in most instances, and definitions of specific CV events may have differed across studies. In many cases, events were not described in published reports or only available in online supplements. However, we made a persistent effort to collect CV outcomes and excluding patients with underlying disease or any other complications.

## Conclusion

In conclusion, the results of this meta-analysis suggest that incretin-based therapy show no significant protective effect on CV events in T2DM primary prevention group with low CV risks.

Current evidence is however, not definitive and associations in prospective long-term safety controlled trials are required to clearly determine the risk/benefit ratio for incretin-based therapies.

## Supporting Information

S1 Fig Risk of bias graph of included trials. (TIF)

S2 Fig Funnel plot of included trials. (TIF)

S3 Fig Subgroup analysis by type of control and mode of therapy. (TIF)

S4 Fig Subgroup analysis by predefined cardiovascular disease. (TIF)

S5 Fig Subgroup analysis by type of incretin. (TIF)

S1 Table Risk of bias assessed of included trials. (PDF)

S2 Table PRISMA checklist. (DOC)
