# Correction: Cardiovascular Effect of Incretin-Based Therapy in Patients with Type 2 Diabetes Mellitus: Systematic Review and Meta-Analysis

**DOI:** 10.1371/journal.pone.0191744

**Published:** 2018-01-19

**Authors:** Je-Yon Kim, Seungwon Yang, Jangik I. Lee, Min Jung Chang

In the Funding and Acknowledgments section, the grant number from the funder Global Center of Excellence in Clinical Trials is listed incorrectly. The correct grant number is: HI14C1062.
