# Supplementary figures and images for: Cardiovascular Effect of Incretin-Based Therapy in Patients with Type 2 Diabetes Mellitus: Systematic Review and Meta-Analysis
Source: PLoS One. 2016 Apr 14;11(4):e0153502. doi: 10.1371/journal.pone.0153502 (PMC4831684; doi:10.1371/journal.pone.0153502)

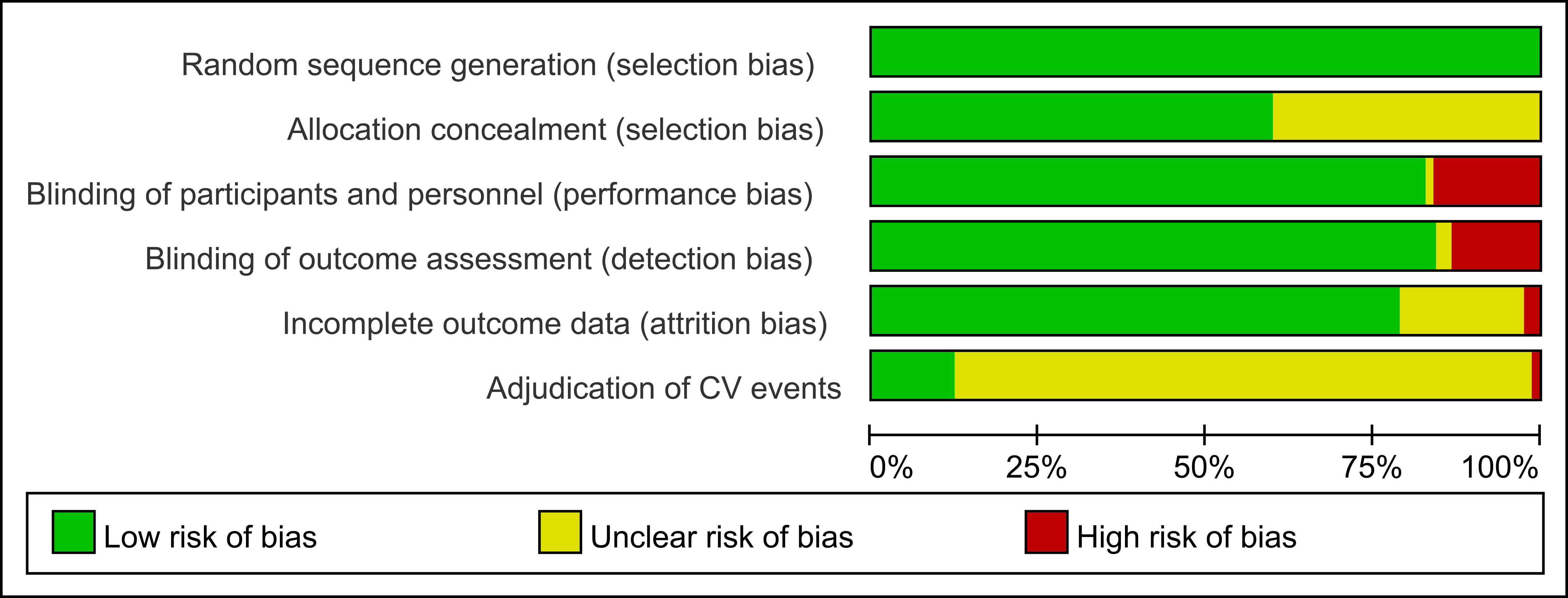

Supplement: S1 Fig — (TIF) [file pone.0153502.s001.tif]

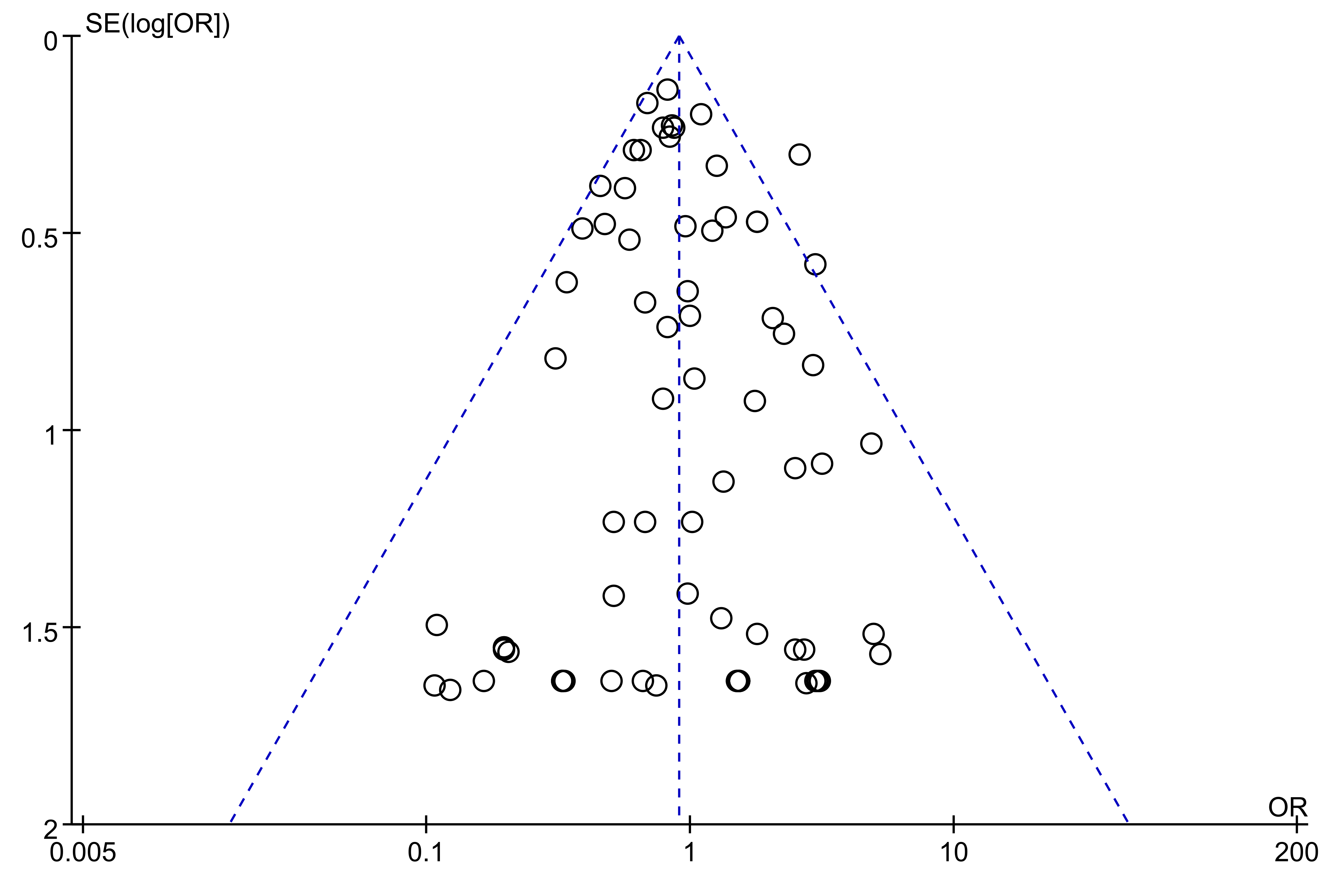

Supplement: S2 Fig — (TIF) [file pone.0153502.s002.tif]

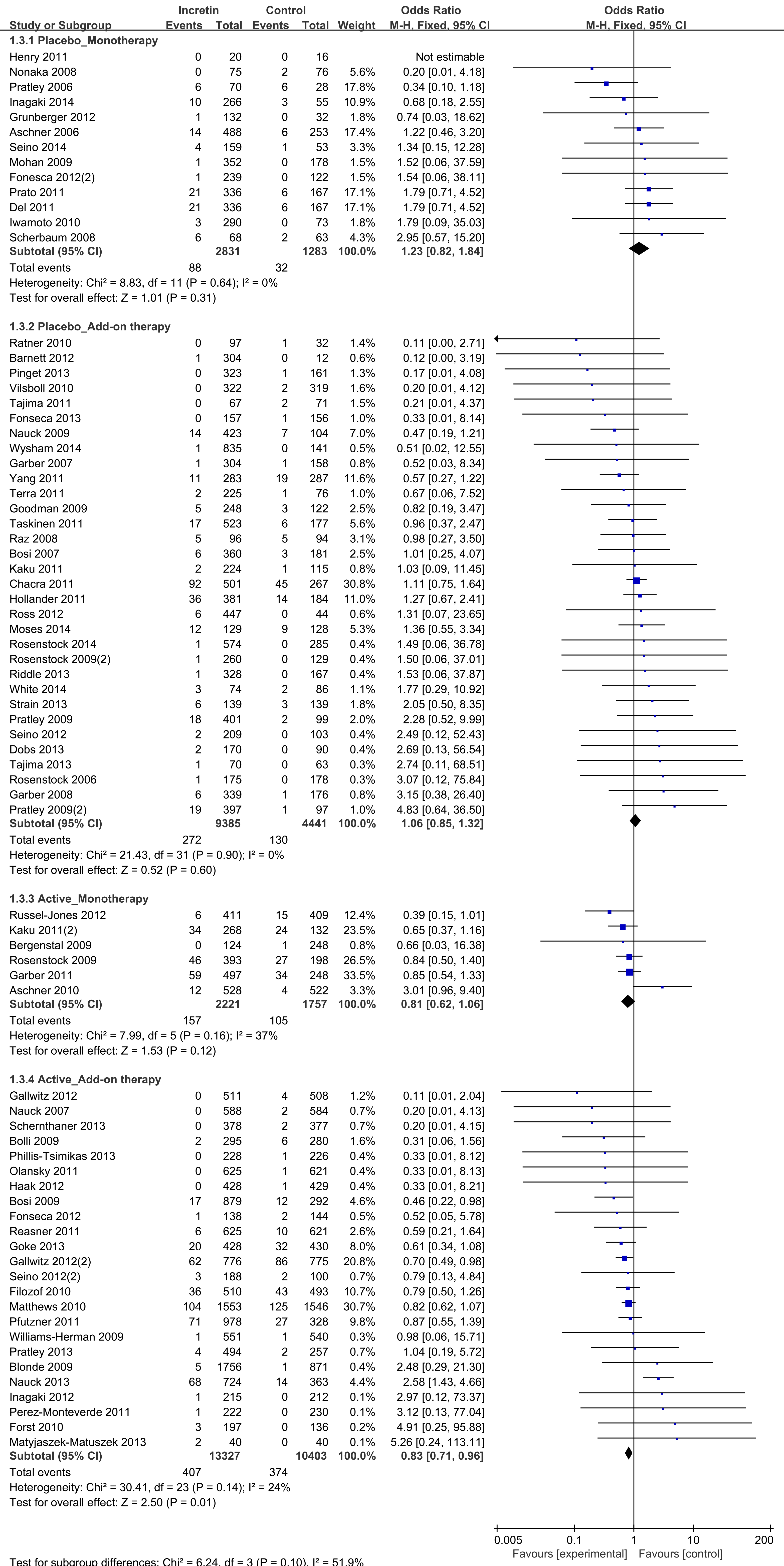

Supplement: S3 Fig — (TIF) [file pone.0153502.s003.tif]

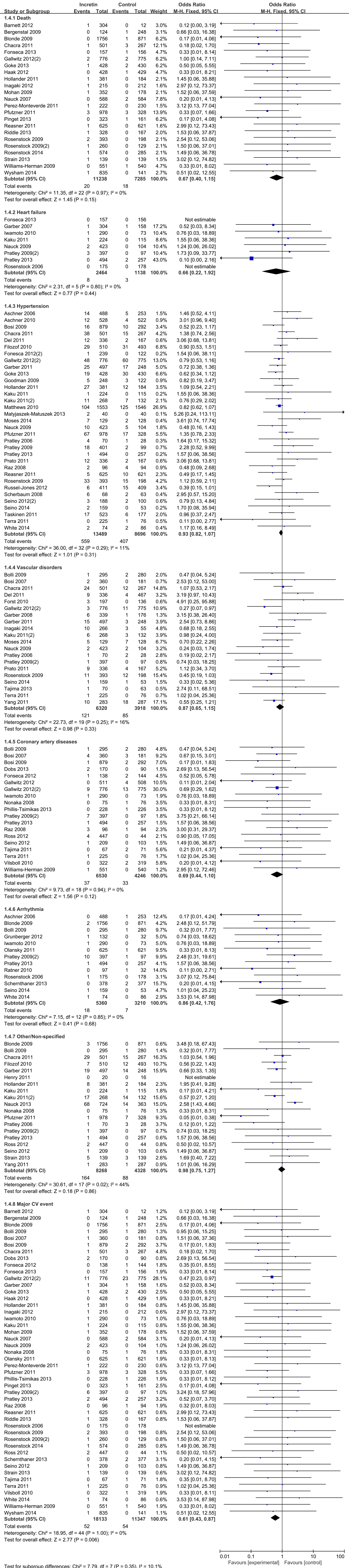

Supplement: S4 Fig — (TIF) [file pone.0153502.s004.tif]

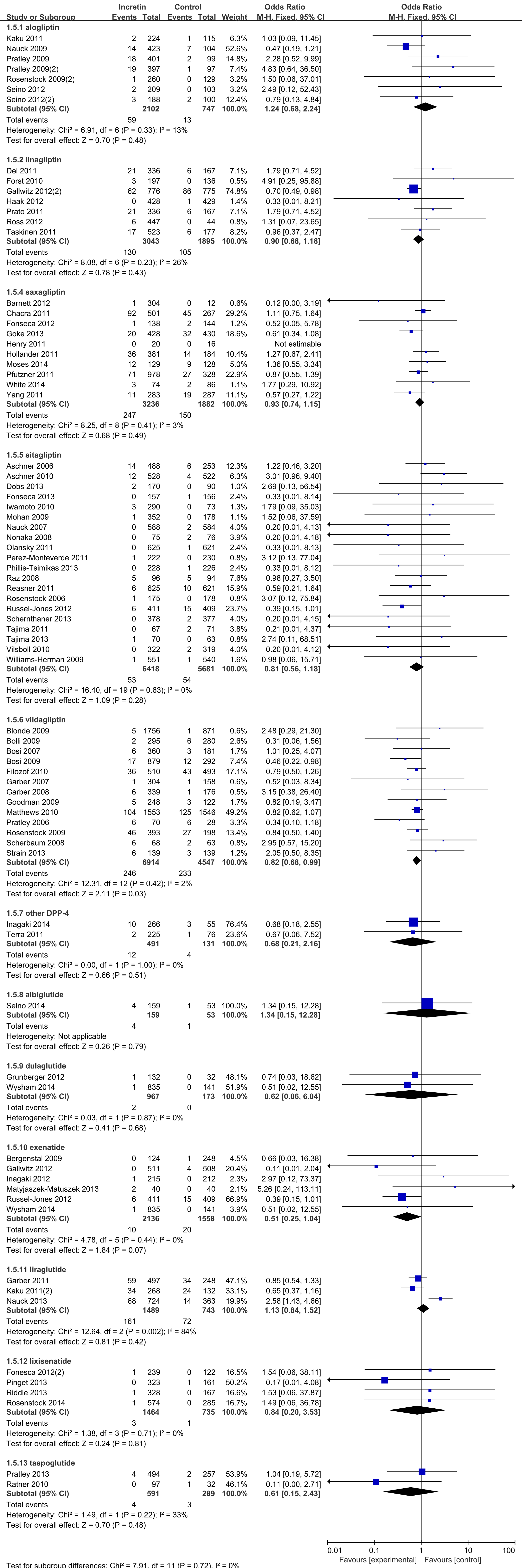

Supplement: S5 Fig — (TIF) [file pone.0153502.s005.tif]
