# Supplementary material for: Cardiovascular Effect of Incretin-Based Therapy in Patients with Type 2 Diabetes Mellitus: Systematic Review and Meta-Analysis
Source: PLoS One. 2016 Apr 14;11(4):e0153502. doi: 10.1371/journal.pone.0153502 (PMC4831684; doi:10.1371/journal.pone.0153502)
Supplement: S1 Table — (PDF) [file pone.0153502.s006.pdf]

S1 Table. Risk of bias assessed of included trials

Author (year)	Random Sequence Generation	Allocation Concealment	Blinding of Participants and Personnel	Blinding of Outcome Assessment	Incomplete Outcome Data	Adjudication of CV Events
Aschner 2006	low	unclear	low	low	low	unclear
Aschner 2010	low	unclear	low	low	unclear	unclear
Barnett 2012	low	low	low	unclear	low	unclear
Bergenstal 2009	low	low	high	high	low	unclear
Blonde 2009	low	low	high	high	unclear	unclear
Bolli 2009	low	low	low	low	unclear	unclear
Bosi 2007	low	unclear	low	low	low	unclear
Bosi 2009	low	unclear	low	low	low	unclear
Chacra 2011	low	unclear	low	low	low	unclear
Del 2011	low	low	low	low	low	unclear
Dobs 2013	low	low	low	low	low	unclear
Filozof 2010	low	unclear	low	low	unclear	low
Fonesca 2012(2)	low	low	low	low	low	unclear
Fonseca 2012	low	low	low	high	low	unclear
Fonseca 2013	low	low	low	low	low	low
Forst 2010	low	low	high	high	low	unclear
Gallwitz 2012	low	low	high	low	high	unclear
Gallwitz 2012(2)	low	low	low	low	low	low
Garber 2007	low	unclear	low	low	unclear	unclear
Garber 2008	low	low	low	low	unclear	unclear
Garber 2011	low	low	high	high	low	unclear
Goke 2013	low	unclear	low	low	unclear	unclear
Goodman 2009	low	unclear	low	low	low	unclear
Grunberger 2012	low	low	low	low	unclear	unclear
Haak 2012	low	unclear	low	low	low	unclear
Henry 2011	low	unclear	low	low	low	unclear
Hollander 2011	low	unclear	low	low	unclear	unclear

Author (year)	Random Sequence Generation	Allocation Concealment	Blinding of Participants and Personnel	Blinding of Outcome Assessment	Incomplete Outcome Data	Adjudication of CV Events
Inagaki 2012	low	low	high	high	low	unclear
Inagaki 2014	low	low	low	low	low	unclear
Iwamoto 2010	low	low	low	low	low	unclear
Kaku 2011	low	low	low	low	low	unclear
Kaku 2011(2)	low	unclear	high	high	low	unclear
Matthews 2010	low	unclear	low	low	low	unclear
Matyjaszek-Matuszek 2013	low	unclear	high	high	unclear	unclear
Mohan 2009	low	low	low	low	low	unclear
Moses 2014	low	low	low	low	low	unclear
Nauck 2007	low	unclear	low	unclear	low	unclear
Nauck 2009	low	low	low	low	low	unclear
Nauck 2013	low	unclear	high	high	low	unclear
Nonaka 2008	low	low	low	low	low	unclear
Olansky 2011	low	unclear	low	low	low	unclear
Perez-Monteverde 2011	low	unclear	low	low	low	unclear
Pfutzner 2011	low	low	low	low	low	unclear
Phillis-Tsimikas 2013	low	low	high	low	low	low
Pinget 2013	low	low	low	low	low	low
Pratley 2006	low	unclear	low	low	low	unclear
Pratley 2009	low	unclear	low	low	low	unclear
Pratley 2009(2)	low	low	low	low	low	low
Pratley 2013	low	low	low	low	low	unclear
Prato 2011	low	low	low	low	low	unclear
Ratner 2010	low	low	low	low	low	unclear
Raz 2008	low	unclear	low	low	low	unclear
Reasner 2011	low	unclear	low	low	low	unclear

Author (year)	Random Sequence Generation	Allocation Concealment	Blinding of Participants and Personnel	Blinding of Outcome Assessment	Incomplete Outcome Data	Adjudication of CV Events
Riddle 2013	low	low	low	low	low	low
Rosenstock 2006	low	unclear	low	low	low	unclear
Rosenstock 2009	low	low	low	low	unclear	unclear
Rosenstock 2009(2)	low	low	low	low	low	unclear
Rosenstock 2014	low	unclear	low	low	low	unclear
Ross 2012	low	low	low	low	low	low
Russel-Jones 2012	low	low	low	low	high	high
Scherbaum 2008	low	unclear	low	low	unclear	unclear
Schernthaner 2013	low	low	low	low	low	unclear
Seino 2012	low	unclear	high	high	unclear	unclear
Seino 2012(2)	low	low	low	low	low	unclear
Seino 2014	low	unclear	low	low	unclear	low
Strain 2013	low	low	low	low	low	unclear
Tajima 2011	low	low	low	low	low	unclear
Tajima 2013	low	low	low	low	low	unclear
Taskinen 2011	low	unclear	low	low	high	unclear
Terra 2011	low	unclear	low	low	low	unclear
Vilsboll 2010	low	low	low	low	low	unclear
White 2014	low	low	low	low	low	unclear
Williams-Herman 2009	low	low	low	low	low	unclear
Wysham 2014	low	low	low	low	low	unclear
Yang 2011	low	unclear	low	low	low	unclear
